# Echocardiographic Evaluation of Right Ventricular (RV) Performance over Time in COVID-19-Associated ARDS—A Prospective Observational Study

**DOI:** 10.3390/jcm10091944

**Published:** 2021-05-01

**Authors:** Golschan Asgarpur, Sascha Treskatsch, Stefan Angermair, Michaela Danassis, Anna Maria Nothnagel, Christoph Toepper, Ralf Felix Trauzeddel, Michael Nordine, Julia Heeschen, Alaa Al-Chehadeh, Ulf Landmesser, Leif Erik Sander, Florian Kurth, Christian Berger

**Affiliations:** 1 Department of Anesthesiology and Intensive Care Medicine, Charité Campus Benjamin Franklin, Corporate Member of Freie Universität and Humboldt Universität zu Berlin, Charité—Universitätsmedizin Berlin, 12203 Berlin, Germany; golschan.asgarpur@charite.de (G.A.); stefan.angermair@charite.de (S.A.); michaela.danassis@charite.de (M.D.); anna-maria.nothnagel@charite.de (A.M.N.); christoph.toepper@charite.de (C.T.); ralf-felix.trauzeddel@charite.de (R.F.T.); michael.nordine@charite.de (M.N.); julia.heeschen@charite.de (J.H.); alaa.al-chehadeh@charite.de (A.A.-C.); christian.berger@charite.de (C.B.); 2 Department of Cardiology, Charité Campus Benjamin Franklin, Corporate Member of Freie Universität and Humboldt Universität zu Berlin, Charité—Universitätsmedizin Berlin, 12203 Berlin, Germany; ulf.landmesser@charite.de; 3 Department of Infectious Disease and Respiratory Medicine, Corporate Member of Freie Universität and Humboldt Universität zu Berlin, Charité—Universitätsmedizin Berlin, 12203 Berlin, Germany; leif-erik.sander@charite.de (L.E.S.); florian.kurth@charite.de (F.K.)

**Keywords:** COVID-19, transthoracic echocardiography, RV function, ARDS, intensive care

## Abstract

(1) Background: To evaluate time-dependent right ventricular (RV) performance in patients with COVID-19-associated acute respiratory distress syndrome (ARDS) undergoing intensive care (ICU) treatment. (2) Methods: This prospective observational study included 21 ICU patients with COVID-19-associated ARDS in a university hospital in 2020 (first wave). Patients were evaluated by transthoracic echocardiography at an early (EE) and late (LE) stage of disease. Echocardiographic parameters describing RV size and function as well as RV size in correlation to P_a_O_2_/F_i_O_2_ ratio were assessed in survivors and nonsurvivors. (3) Results: Echocardiographic RV parameters were within normal range and not significantly different between EE and LE. Comparing survivors and nonsurvivors revealed no differences in RV performance at EE. Linear regression analysis did not show a correlation between RV size and P_a_O_2_/F_i_O_2_ ratio over all measurements. Analysing EE and LE separately showed a significant increase in RV size correlated to a lower P_a_O_2_/F_i_O_2_ ratio at a later stage of COVID-19 ARDS. (4) Conclusion: The present study reveals neither a severe RV dilatation nor an impairment of systolic RV function during the initial course of COVID-19-associated ARDS. A trend towards an increase in RV size in correlation with ARDS severity in the second week after ICU admission was observed.

## 1. Introduction

The 2019 Coronavirus disease (COVID-19) caused by the novel SARS Coronavirus 2 (SARS-CoV-2) is responsible for a global pandemic, infecting millions of people worldwide. In certain cases, a fulminant SARS-CoV-2 infection led to severe acute respiratory distress syndrome (ARDS), as well affecting other organ systems, such as the nervous and the cardiovascular system [1,2]. The incidence of hospitalized patients with COVID-19 developing ARDS is approximately 33% [3] and is associated with poor clinical outcome and a high mortality rate, reaching up to 45% according to recent research [4].

The pathophysiology of non-COVID-associated ARDS has been described as an increased permeability to liquid, protein and cellular compounds across the lung endothelium, leading to interstitial edema, which further translocate across the alveolar barrier into the alveolar space, impairing oxygenation [5]. The resulting hypoxemia caused by a ventilation-to-perfusion mismatch as well as right-to-left intrapulmonary shunting provokes compensatory hypoxic pulmonary vasoconstriction (HPV) with consecutive pulmonary hypertension and increased afterload, leading to right ventricular (RV) impairment or failure [6,7]. Non-COVID ARDS-associated RV failure is associated with a mortality rate of up to 60–70% [2,6,8].

Virus-associated RV impairment has been documented during the outbreak of H1N1 in 2009 [9]. However, in contrast to the pathophysiological course of non-COVID ARDS, early experiences with COVID-19-associated ARDS raised the suspicion for a loss of pulmonary vascular tone. Therapeutic strategies aiming to dilate alveolar pulmonary vessels in the area of alveoli still participating in gas exchange in order to improve gas exchange, with for example, inhaled nitric oxide, were found to be not as clinically effective as deemed [10]. Thus, we hypothesized that hypoxemia in COVID-19-associated ARDS may present as a different hemodynamic phenotype without increases in afterload, thus impairing RV performance. To investigate this hypothesis, we evaluated time-dependent RV performance in COVID-19-associated ARDS intensive care (ICU) patients using transthoracic echocardiography (TTE) as a noninvasive diagnostic and readily available bedside tool [7,8].

## 2. Materials and Methods

For evaluating the impact of COVID-19 ARDS on RV performance, we conducted a monocenter prospective observational study at the department of anesthesiology and intensive care medicine, Campus Benjamin Franklin, Charité—Universitätsmedizin Berlin. This study was constituted as a substudy of the PA-COVID-19 trial [11], approved by Charité’s Ethics committee (EA2/066/20). Informed consent was obtained from all participants or their authorized representative. The trial was registered with the following number DRKS00021688 on 13 May 2020 (WHO International Clinical Trials Registry Platform).

Adult patients with positive SARS-CoV-2 polymerase chain reaction (PCR) testing admitted to our intensive care unit (ICU) requiring treatment due to COVID-19 ARDS in accordance with the Berlin Definition [4] were included in this study during the first wave in Germany in 2020. Patients with a palliative approach to therapy were excluded. All participants were scheduled for an early TTE evaluation (EE) of RV performance within the first week of ICU treatment. For evaluating a possible time-dependent impact on RV performance, an additional “late” transthoracic echocardiography (LE) was conducted in the second week after ICU admission. All TTE examinations were performed by echocardiography-trained ICU practitioners according to national standards [12] and actual guidelines [13]. For evaluation of RV performance, the 2-dimensional apical-4-chamber (A4C) or subcostal 4-chamber view (SC4C) were obtained using a VIVID S60 ultrasound system (GE Healthcare, Chicago, IL, USA). The following parameters were analyzed using EchoPac (GE Healthcare, Chicago, USA) according to recent guidelines: RV medial diameter (RVMD), RV end diastolic area index (RVEDAi), RV/LV medial diameter ratio (RLDR), RV/LV area ratio (RLAR), tricuspid annular plain systolic excursion (TAPSE) and RV fractional area change (RFAC) [13].

Demographic, morphometric, laboratory, respiratory/ventilatory and hemodynamic data were obtained from two patient data management systems (COPRA System GmbH, Sasbachwalden, Germany and SAP AG, Walldorf, Germany) at two time points: EE and LE. All data are available on demand.

Descriptive analyses and statistical testing were performed using IBM SPSS Statistics (version 25; IBM, Armonk, NY, USA) with a *p*-value below 0.05 regarded as significant. Unless otherwise stated, all data are presented as median and interquartile range (IQR). Statistical significance among groups was analyzed by the exact nonparametric Mann–Whitney U test or Wilcoxon single rank test. Exact chi-square tests were used for qualitative data. Linear regression (r^2^) was calculated to detect correlations between parameters describing RV performance and oxygenation index as surrogate for ARDS severity. All tests should be understood as constituting explorative analysis, and no adjustment for multiple testing was performed.

## 3. Results

**Figure 1:**
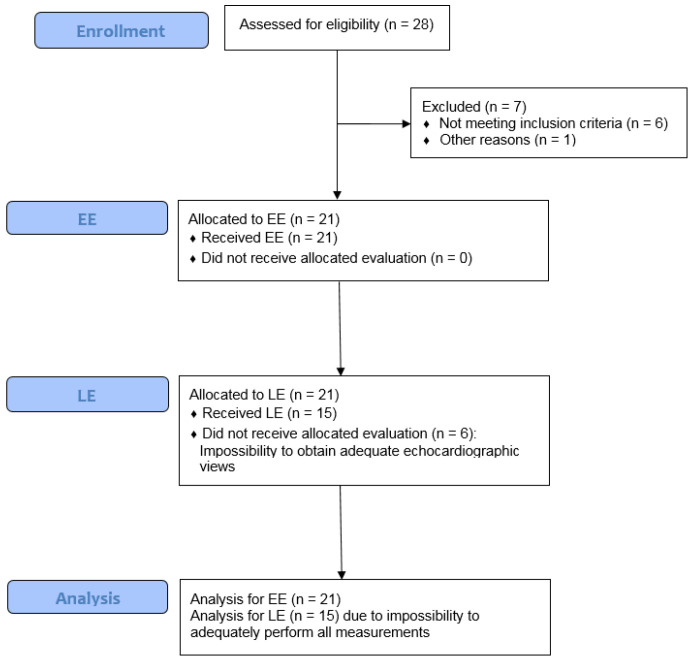
Consort flow diagram.

Between March and May 2020, 28 patients were assessed for eligibility. Twenty-one patients were included in this study, while six did not meet inclusion criteria (no ARDS according to Berlin definition), and one patient was transferred to a different ICU after initial evaluation (Figure 1).

Median (IQR) age of all included patients was 68 (59/76) years with a body mass index (BMI) of 27.8 (24.0/31.8) kg/m^2^ and a gender distribution of 9/12 (female/male). Seven out of 21 patients died, accounting for a mortality rate of 33%. Median time from ICU admission to death was 20 (12/30) days. Detailed morphometric and demographic data of survivors and nonsurvivors at ICU admission are presented in Table 1. Except for hyperlipoproteinemia (HLP), which found was more amongst nonsurvivors (survivors 7%, nonsurvivors 43%; *p* = 0.007), both groups exhibited comparable age, gender, BMI and comorbidities (for detailed and patient individual information for comorbidities, see Supplementary Table S1). Laboratory parameters at ICU admission showed no differences between survivors and nonsurvivors (Supplemental Table S2).

Increased APACHE II and SAPS II scores at ICU admission depict an increased disease severity in nonsurvivors despite comparable ratios of arterial partial pressure of oxygen to fraction of inspired oxygen (P_a_O_2_/F_i_O_2_) (Table 2).

Seventeen out of 21 patients (81%) required invasive mechanical ventilation at ICU admission (survivors: *n* = 11; nonsurvivors *n* = 6) (Table 2), and nearly all patients had bacterial superinfections during ICU treatment (Supplementry Table S2). Time from ICU admission to EE was 1.7 (0.4/3.7) days and 11.0 (7.9/12.8) days to LE. No patient died before the LE examination. Clinical conditions were comparable between EE and LE (Table 3) as well as between survivors and nonsurvivors at EE (Table 4). Computer tomography (CT) confirmed segmental pulmonary embolism (PE) in 5 of 21 (24%) patients during the observation period without significant differences between survivors (*n* = 3; 21%) and nonsurvivors (*n* = 2; 29%) (*p* = 0.717).

Echocardiographic parameters evaluating RV size (RVMD, RVEDAi, RLDR, RLAR) and systolic function (TAPSE, RFAC) were not significantly different between both time points (Table 5). Comparing survivors with nonsurvivors also revealed no differences in RV performance parameters at EE (Table 6). Paradoxical movement of the interventricular septum (IVS) was observed in only one patient at EE.

**Figure 2:**
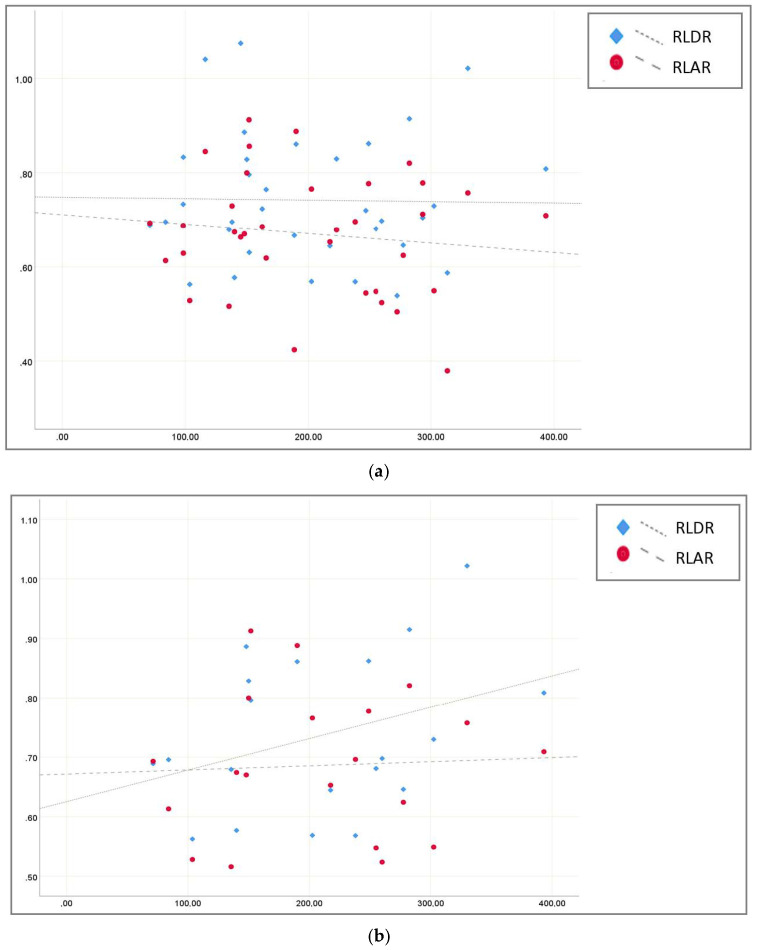

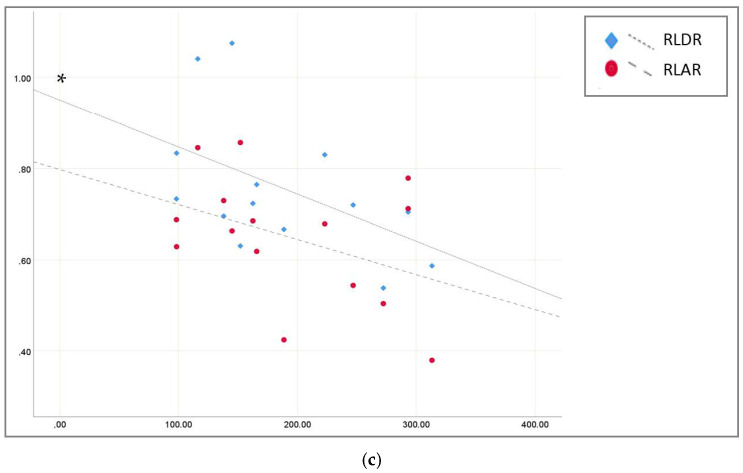
(**a**): Linear regression of RV/LV ratio (RLAR = red; RLDR = blue) and pO_2_/F_i_O_2_ ratio; All measurements (RLDR: r^2^ = 0.000, RLAR: r^2^ = 0.02); (**b**): Linear regression of RV/LV ratio (RLAR = red; RLDR = blue) and pO_2_/F_i_O_2_ ratio EE (RLDR: r^2^ _=_ 0.149, RLAR: r^2^ = 0.002); (**c**): Linear regression of RV/LV ratio (RLAR = red; RLDR = blue) and pO_2_/F_i_O_2_ ratio LE (RLDR r^2^ = 0.27 *, RLAR r^2^ = 0.168); * = *p* < 0.05.

Linear regression analysis including all echocardiographic measurements did not detect a correlation between RV size and P_a_O_2_/F_i_O_2_ ratio (RLDR: r^2^ = 0.000, *p* = 0.907; RLAR: r^2^ = 0.02, *p* = 0.466) (Figure 2a). Separate evaluation of time-dependent RV performance also found no correlation between RV size and P_a_O_2_/F_i_O_2_ ratio at EE (RLDR: r^2^ _=_ 0.149, *p* = 0.084; RLAR: r^2^ = 0.002, *p* = 0.835) (Figure 2b). Nevertheless, a trend towards a slight, though significant increase in RV size associated with a decreased P_a_O_2_/F_i_O_2_ ratio became noticeable in the second week of ICU treatment at LE (RLDR: r^2^ = 0.27, *p* = 0.047; RLAR: r^2^ = 0.168, *p* = 0.129) (Figure 2c).

## 4. Discussion

This observational study is the first to evaluate the time-dependent impact of COVID-19-associated ARDS on RV performance and demonstrated a virtually unaffected RV with absence of severe RV dilatation within the first two weeks after ICU admission using two-dimensional echocardiography. We were also unable to detect any discernible RV differences between survivors and nonsurvivors during the observation period via echocardiographic bedside examinations. Although RV values remained within normal or acceptable ranges, according to proposed values in COVID-19 ARDS, non-COVID ARDS and/or ASE/ESC guidelines [13,14,15], a trend towards RV enlargement correlating with a decrease in P_a_O_2_/F_i_O_2_ ratio—as surrogate marker for ARDS severity—was observed during the second week of evaluation. These results may support previous assumptions that COVID-19 ARDS may present as an altered hemodynamic phenotype without severe impairment of RV performance.

RV dilatation or dysfunction in non-COVID ARDS is understood as a result of physiological changes in the pulmonary circulation [16]. The mechanism of acute cor pulmonale in non-COVID ARDS has been established as refractory pulmonary edema due to endothelial cell swelling and hypoxemia, leading to HPV in precapillary arterioles and consecutively increasing RV afterload. Additional microvascular thrombosis deriving from endothelial cell activation can further promote RV deterioration, and later remodeling can lead to persistent pulmonary hypertension [17,18].

According to this pathophysiological pathway, we assumed that RV function during COVID-19-associated ARDS would follow suit, however, we were not able to detect any meaningful RV impairment in COVID-19 ARDS patients in our results. For higher accuracy, we determined RV size not only in terms of absolute diameter, but also in correlation to LV size using two different methods: end-diastolic RV/LV diameter (RLDR) and end-diastolic RV/LV area (RLAR). With a calculated RLDR and RLAR between 0.68 and 0.72 in conjunction with a preserved systolic RV function, our COVID-19 ARDS cohort revealed, at most, only a slight increase in RV size. Whether or not this increase implies a clinically relevant RV impairment remains controversial, as former studies regarding disease impact on RV set a much higher limit for a relevant RV/LV ratio increase [19,20,21]. Analyzing timed-dependent RV performance in our ICU cohort, an absence of severe RV impairment was also observed at a later stage of disease. Further, to investigate if a more severe COVID-19 ARDS may correlate with a pronounced RV impairment, we analyzed linear regression of P_a_O_2_/F_i_O_2_ ratios with RV/LV ratios. Based on experience from non-COVID ARDS pathophysiology, a more severe ARDS should lead to a higher RV/LV ratio. Again, this assumption could not be confirmed in our COVID-19 ARDS cohort as we observed only a slight increase in RV in relation to LV size correlating with a more impaired oxygenation at a later stage of disease.

Interestingly, critically ill patients suffering from H1N1 infection frequently exhibit RV dilatation and failure [9], whereas a recent study showed a reduced incidence of RV failure in ventilated COVID-19 patients [22]. These findings may reflect the varying clinical manifestation due to different pathophysiological changes in non-COVID and COVID-19 ARDS. The precise pathological mechanism in COVID-19 ARDS is still unknown, but comparisons to other viral infections support the hypothesis of a different clinical phenotype with altered pulmonary vascular reactivity [22,23] promoting increased vascular permeability [24] finally leading to vasoplegia [10]. Impaired endothelial function without HPV may thus provide an explanation for the sustained absence of severe RV dysfunction among the COVID-19 ARDS population over time, as demonstrated by our findings.

In this context, the course of COVID-19 as well as non-COVID ARDS may be assumed as being possibly bimodal in nature, or as some authors have suggested, with different phenotypes [10,25]. This theory may be supported by our observed shift to a slightly higher correlation of increased RV/LV ratio with a decreased P_a_O_2_/F_i_O_2_ ratio in the second week after ICU admission. Furthermore, it should be taken into consideration that during the course of virally induced ARDS, bacterial superinfection is a common complication [22,26]. This was also present in our study population, where 80.9% of patients had a proven bacterial superinfection during their ICU treatment (Supplementary Table S3). Hence, it cannot be ruled out that the ARDS phenotype may change over time. It is thus reasonable that the later course of COVID-19 ARDS may present as a mixture of viral and bacterial ARDS, which may be reflected in the observed change in linear regression analysis at LE. The destruction of pulmonary tissue, which we frequently observed in our COVID-19 ARDS (Supplemental Figure S1), may provide another explanation for our findings, especially in the late course. Such destruction may lead to a rarefication of the pulmonary vascular bed resulting in an increased RV afterload. Another possible explanation for a change in RV performance over time may be pulmonary embolism with consecutive RV impairment. Segmental PE was detected in five (21%) patients during the observation period, which is within the reported incidence of PE in COVID-19 [27]. No central PE occurred among our population and comparison of RV parameters in PE and non-PE patients revealed no significant differences in RV performance (Supplementary Table S4). Therefore, additional PE seems to be unlikely to have a relevant impact on the findings in this study.

Only limited data for RV performance in COVID-19 ARDS exist to date and the studies vary in their finding. Other than the above-mentioned pathophysiological theories, some methodical explanations may account for these differences. Recently presented results for RV function in COVID-19 were obtained from single, inconsistent time points among COVID-19 infected patients but without differentiation between presence or absence of ARDS or sepsis [28,29,30,31]. Other working groups have presented data with partly mechanically ventilated COVID-19 patients (30%) [29] or exclusion of ventilated COVID-19 patients [30], while echocardiographic examinations were performed. Another work from Bagate et al. reported at a single time point—with a focus on filling pressures—a high incidence of cor pulmonale, but RV functional parameters like TAPSE were found to be comparable to our findings [28]. These different approaches may lead to a heterogeneity of studied populations and may impact RV evaluation findings in COVID-19 patients. Therefore, we assessed RV performance in every patient, irrespective of ventilation status at an early state after onset of COVID-19-associated ARDS followed by a second evaluation within the second week of treatment. Nevertheless, we did not find any differences in parameters describing sole RV performance as well as in correlation with ARDS severity between survivors and nonsurvivors. The observed slight but not significant decrease of TAPSE in nonsurvivors is somewhat in contrast to a recent study from D’Alto and colleagues. They investigated TAPSE in relation to echocardiographic estimations of systolic pulmonary arterial pressure (sPAP) among a comparable cohort of mostly mechanically ventilated COVID-19 ARDS patients [32]. A significant reduction in TAPSE and TAPSE/sPAP ratio between survivors and nonsurvivors was observed, but despite statistical significance, TAPSE in nonsurvivors was also still within normal ranges confirming our results. In addition, a significant increase in C-reactive protein and procalcitonin were observed only among nonsurvivors in their study, which could be interpreted as a higher incidence of bacterial superinfections at the time of RV assessment. This finding cannot be confirmed by our results as most patients suffered bacterial superinfections irrespective of survival.

This study has some limitations. Due to the course of ARDS, with the need of invasive ventilation, including high PEEP and periodic change to prone position, obtaining adequate TTE views at exactly the same time point was not possible. Further, TTE evaluation in such patients is challenging, therefore, our results are somewhat limited due to the absence of sPAP evaluation. The assessment of RV function via TTE amongst ARDS patients has been observed to be inferior to transesophageal echocardiography (TEE), as TTE is prone to interference due to anatomical barriers such as the chest wall [33]. Additionally, Evrard et al. found that cardiac assessment via TTE was suboptimal compared with TEE assessment amongst COVID-19 patients, and the primary reason for this is the ability to obtain short axis cardiac images with TEE [22]. Because of the increased complexity to obtain proper Doppler-derived sPAP in a positive pressure ventilated ARDS population, we decided to utilize a simple and reproducible protocol for TTE evaluation over time. Furthermore, all participants required (non)invasive ventilatory support at the time of RV evaluation. It may be reasonable to suggest that this intervention improved P_a_O_2_/F_i_O_2_ ratios, thereby preserving RV performance. In this context a baseline TTE evaluation before initiation of ARDS therapy would have been beneficial, but ethically not feasible. Because of the nature of this study, a previous sample size calculation was not possible. Additionally, due to the small sample size in this study, drawing broader conclusions concerning RV function in the majority of COVID-19 patients may be limited. A higher number of cases might have led to more statistically significant differences between groups, as well as increasing the general power of the study.

## 5. Conclusions

Our work reveals neither severe RV dilatation nor impairment of systolic RV function during the initial course of COVID-19-associated ARDS. In contrast to the initial evaluation, a trend towards an increase in RV size in correlation with ARDS severity in the second week after ICU admission was detectable. These findings support the assumptions of impaired regulation of pulmonary vascular and/or endothelial dysfunction due to SARS-CoV-2 and hint towards a change of the COVID-19-associated ARDS phenotype over time. However, final evidence for these assumptions is still missing, and further research into the hemodynamic changes during the course of COVID-19 ARDS needs to be performed.

## Figures and Tables

**Table 1:** Survivor and nonsurvivor demographic data and medical history at intensive care unit (ICU) admission.

	Survivor *n* = 14	Nonsurvivor *n* = 7	*p*
Age (years)	70 (57/76)	68 (62/79)	0.36
Gender (female/male)	7/7	2/5	0.076
BMI (kg/m^2^)	28.2 (23.0/31.7)	27.0 (26.1/34.0)	0.799
History of			
CAD	3 (21%)	0 (0%)	n.a.
AHT	8 (57%)	3 (43%)	0.280
HF	0 (0%)	1 (14%)	n.a.
COPD	1 (7%)	1 (14%)	0.445
IDDM	1 (7%)	0 (0%)	n.a.
NIDDM	2 (14%)	1 (14%)	1.0
CKD	1 (7%)	1 (14%)	0.445
CLD	0 (0%)	0 (0%)	n.a.
HLP	1 (7%)	3 (43%)	0.007
PVD	1 (7%)	0 (0%)	n.a.

BMI: body mass index; CAD: coronary artery disease; AHT: arterial hypertension; HF: heart failure; COPD: chronic obstructive pulmonary disease; IDDM: insulin-dependent diabetes mellitus; NIDDM: non-insulin-dependent diabetes mellitus; CKD: chronic kidney disease; CLD: chronic liver disease; HLP: hyperlipoproteinemia; PVD: peripheral vascular disease.

**Table 2:** Survivor and nonsurvivor medical conditions at ICU admission.

	Survivor *n* = 14	Nonsurvivor *n* = 7	*p*
P_a_O_2_/F_i_O_2_ ratio	126 (98/163)	142 (81/173)	1.00
NIV/IV	3/11	1/6	0.445
SOFA	5 (4/11)	10 (7/12)	0.197
APACHE II	15 (7/19)	21 (11/30)	0.031
SAPS II	31 (22/44)	64 (30/71)	0.02

P_a_O_2_: arterial partial pressure of oxygen, F_i_O_2_: fraction of inspired oxygen, NIV: noninvasive ventilation, IV: invasive ventilation, SOFA: Sequential Organ Failure Assessment, APACHE: Acute Physiology And Chronic Health Evaluation, SAPS: Simplified Acute Physiology Score.

**Table 3:** Scores, respiratory/ventilatory and hemodynamic data obtained at EE and LE echocardiographic examination.

	EE	LE	*p*	No. of Patients
P_a_O_2_/F_i_O_2_ ratio	203 (138/269)	166 (138/273)	0.570	21/15
Invasive Ventilation				
ΔP [mbar]	10.0 (9.5/14)	10.0 (8.5/15.0)	0.929	17/14
PEEP [mbar]	14.0 (10.0/16.0)	14.0 (12.3/15.3)	0.330	17/14
TV [mL/kg]	5.8 (5.4/6.5)	5.9 (4.6/7.1)	0.859	17/14
PaCO2 [mbar]	38 (34/46)	39 (32/46)	0.776	21/15
MAP [mmHg]	70 (65/85)	70 (70/85)	0.653	21/15
HR [BPM]	82 (72/93)	78 (74/103)	0.233	21/15
Norepinephrine [µg/kg/min]	0.05 (0.0/0.17)	0.02 (0.00/0.08)	0.054	21/15
APACHE II	23 (18/28)	28 (21/34)	0.100	21/15
SOFA	9 (4/12)	10 (8/12)	0.059	21/15
SAPS II	40 (37/61)	58 (41/73)	0.139	21/15

P_a_O_2_: arterial partial pressure of oxygen, F_i_O_2_: fraction of inspired oxygen, ΔP: change in mechanical respiratory pressure, PEEP: positive end expiratory pressure, TV: tidal volume, MAP: mean arterial pressure, HR: heart rate, SOFA: Sequential Organ Failure Assessment, APACHE: Acute Physiology And Chronic Health Evaluation, SAPS: Simplified Acute Physiology Score.

**Table 4:** Respiratory/ventilator, hemodynamic data, and clinical scores at EE for survivors and nonsurvivors.

	Survivors	Nonsurvivors	*p*	No. of Patients
P_a_O_2_/F_i_O_2_ ratio	177 (128/256)	238 (148/283)	0.535	14/7
Invasive Ventilation				
ΔP [mbar]	11 (9/14)	10 (10/15)	0.961	11/6
PEEP [mbar]	14 (10/15)	16 (13/17)	0.256	11/6
TV [mL/kg]	6.0 (5.4/6.9)	5.6 (5.1/6.8)	0.428	11/6
PaCO2 [mbar]	36 (31/41)	48 (37.0/50.0)	0.036	14/7
MAP [mmHg]	73 (65/85)	70 (65/85)	0.799	14/7
HR [BPM]	81 (70/91)	90 (72/95)	0.360	14/7
Norepinephrine [µg/kg/min]	0.04 (0.0/0.13)	0.13 (0.0/0.22)	0.360	14/7
APACHE II	20 (17/27)	28 (21/29)	0.197	14/7
SOFA	8 (4/11)	10 (6/12)	0.172	14/7
SAPS II	40 (36/55)	56 (38/65)	0.287	14/7

P_a_O_2_: arterial partial pressure of oxygen, F_i_O_2_: fraction of inspired oxygen, ΔP: driving pressure, PEEP: positive end expiratory pressure, TV: tidal volume, MAP: mean arterial pressure, HR: heart rate, SOFA: Sequential Organ Failure Assessment, APACHE: Acute Physiology And Chronic Health Evaluation, SAPS: Simplified Acute Physiology Score.

**Table 5:** TTE evaluated parameters for right ventricular function at EE and LE.

	EE	LE	*p*	No. of Patients
RVMD [mm]	30 (28/37)	32 (29/37)	0.724	21/15
RVEDAi	11.6 (9.8/13.0)	12.4 (10.8/13.5)	0.820	20/15
RLDR	0.70 (0.63/0.84)	0.72 (0.67/0.81)	0.570	21/15
RLAR	0.68 (0.57/0.77)	0.68 (0.54/0.73)	0.191	20/15
TAPSE [mm]	22 (19/26)	24 (21/27)	0.345	19/15
RFAC [%]	0.35 (0.30/0.44)	36.0 (32.9/39.7)	0.650	20/15

RVMD: right ventricular medial diameter, RVEDAi: RV enddiastolic area index, RLDR: RV/LV diameter ratio, RLAR: RV/LV area ratio, TAPSE: tricuspid annular plain systolic excursion, RFAC: RV fractional area change.

**Table 6:** TTE evaluated parameters for right ventricular function, survivors compared to nonsurvivors at EE.

	Survivors	Nonsurvivors	*p*	No. of Patients
RVMD [mm]	31 (29/37)	28 (25/39)	0.360	14/7
RVEDAi	12.0 (10.0/14.2)	10.7 (9.7/12.5)	0.397	14/6
RLDR	0.69 (0.63/0.81)	0.73 (0.62/0.89)	0.689	14/7
RLAR	0.68 (0.54/0.77)	0.68 (0.61/0.84)	0.602	14/6
TAPSE [mm]	23 (20/26)	21 (19/25)	0.579	13/6
RFAC [%]	35 (32/41)	36 (25/47)	0.779	14/6

RVMD: right ventricular medial diameter, RVEDAi: RV enddiastolic area index, RLDR: RV/LV diameter ratio, RLAR: RV/LV area ratio, TAPSE: tricuspid annular plain systolic excursion, RFAC: RV fractional area change.

## Data Availability

The data presented in this study are available on request from the corresponding author. The data are not publicly available due to ethical restrictions.

## Supplementary Materials

The following are available online at https://www.mdpi.com/article/10.3390/jcm10091944/s1, Table S1: Known medical history, Table S2: Laboratory data on ICU admission from survivors vs. nonsurvivors, Table S3: Presumed superinfections and proven superinfections, pulmonary and extrapulmonary during ICU stay, Table S4: TTE evaluated parameters for right ventricular function at EE in patients without PE compared to patients with PE, Figure S1: Pat. No 4, CT Scan of the lung at LE, after first week of ICU stay due to COVID-19 ARDS (mechanically ventilated).
