# Supplementary material for: Echocardiographic Evaluation of Right Ventricular (RV) Performance over Time in COVID-19-Associated ARDS—A Prospective Observational Study
Source: J Clin Med. 2021 May 1;10(9):1944. doi: 10.3390/jcm10091944 (PMC8125118; doi:10.3390/jcm10091944)
Supplement: Supplementary file 1 [file jcm-10-01944-s001.zip › jcm-1162605-SI.pdf]

	PATIENT NO.	KNOWN HISTORY OF									
		CAD	AHT	HF	COPD	IDDM	NIDDM	CKD	CLD	HLP	PVD
SURVIVED	1	No	No	No	No	No	No	No	No	No	No
	2	No	No	No	No	No	No	No	No	No	No
	3	No	YES	No	No	YES	No	YES	No	YES	No
	4	No	YES	No	No	No	No	No	No	No	No
	5	No	No	No	No	No	No	No	No	No	No
	6	No	YES	No	No	No	No	No	No	No	No
	7	YES	YES	No	No	No	No	No	No	No	No
	8	YES	No	No	No	No	No	No	No	No	No
	9	No	No	No	No	No	No	No	No	No	No
	10	No	No	No	YES	No	No	No	No	No	No
	11	No	YES	No	No	No	YES	No	No	No	No
	12	YES	YES	No	No	No	No	No	No	No	YES
	13	No	YES	No	No	No	YES	No	No	No	No
	14	No	YES	No	No	No	No	No	No	No	No
DECEASED	15	No	No	No	No	No	No	No	No	No	No
	16	No	No	No	No	No	No	No	No	No	No
	17	No	YES	No	No	No	No	No	No	No	No
	18	No	YES	No	No	No	No	No	No	YES	No
	19	No	No	YES	No	No	No	YES	No	YES	No
	20	No	No	No	No	No	No	No	No	No	No
	21	No	YES	No	YES	No	YES	No	No	YES	No

Supplemental Table 1: known medical history (CAD: coronary artery disease; AHT: arterial hypertension; HF: heart failure; COPD: chronic obstructive pulmonary disease; IDDM: insulin dependent diabetes mellitus; NIDDM: non-insulin dependent diabetes mellitus; CKD: chronic kidney disease; CLD: chronic liver disease; HLP: hyperlipoproteinemia; PVD: Peripheral vascular disease)

	Survivor (n=14)	Non-Survivor (n=7)	P
Leucocytes (no/nl)	8.6 (5.4/14.9)	7.2 (5.1/12.3)	0.689
Lymphocytes (no/nl)	0.85 (0.43/1.41)	0.75 (0.39/1.05)	0.585
Thrombocytes (no/nl)	194 (132/285)	188 (143/254)	1.0
Haemoglobine (g/dl)	12.7 (11.2/14.1)	11.7 (10.5/13.4)	0.856
Creatine kinase (U/l)	140 (46/546)	223 (40/396)	0.757
C-reactive protein (mg/l)	126 (53/209)	144 (52/334)	0.488
Lactate dehydrogenase (U/l)	428 (301/558)	397 (300/406)	0.535
Creatinine (mg/dl)	0.86 (0.70/1.14)	1.29 (0.97/1.75)	0.056
Billirubin, total (mg/dl)	0.62 (0.31/0.73)	0.52 (0.39/1.10)	0.689
Lactate (mg/dl)	10.5 (8.0/19.3)	9.0 (8.0/16.0)	0.743
Prothrombin time (%)	73 (46/93)	76 (63/100)	0.322

Supplemental table 2: Laboratory data on ICU admission from survivors vs. non-survivors

PATIENT NO.		SUSPECTED/PROVEN SUPERINFECTION		
		**presumed superinfection**	**proven pulmonal bacterial infection**	**proven extrapulmonal bacterial infection**
SURVIVED	1	YES	No	No
	2	YES	No	No
	3	YES	No	YES
	4	YES	YES	YES
	5	No	No	YES
	6	YES	YES	YES
	7	YES	YES	YES
	8	YES	YES	YES
	9	YES	YES	No
	10	YES	YES	YES
	11	YES	YES	YES
	12	YES	YES	YES
	13	YES	YES	YES
	14	YES	No	No
DECEASED	15	YES	No	YES
	16	YES	No	YES
	17	No	No	No
	18	YES	No	YES
	19	YES	YES	YES
	20	YES	YES	YES
	21	YES	No	YES

Supplemental table 3: Presumed superinfections and proven superinfections, pulmonary and extrapulmonary, during ICU stay

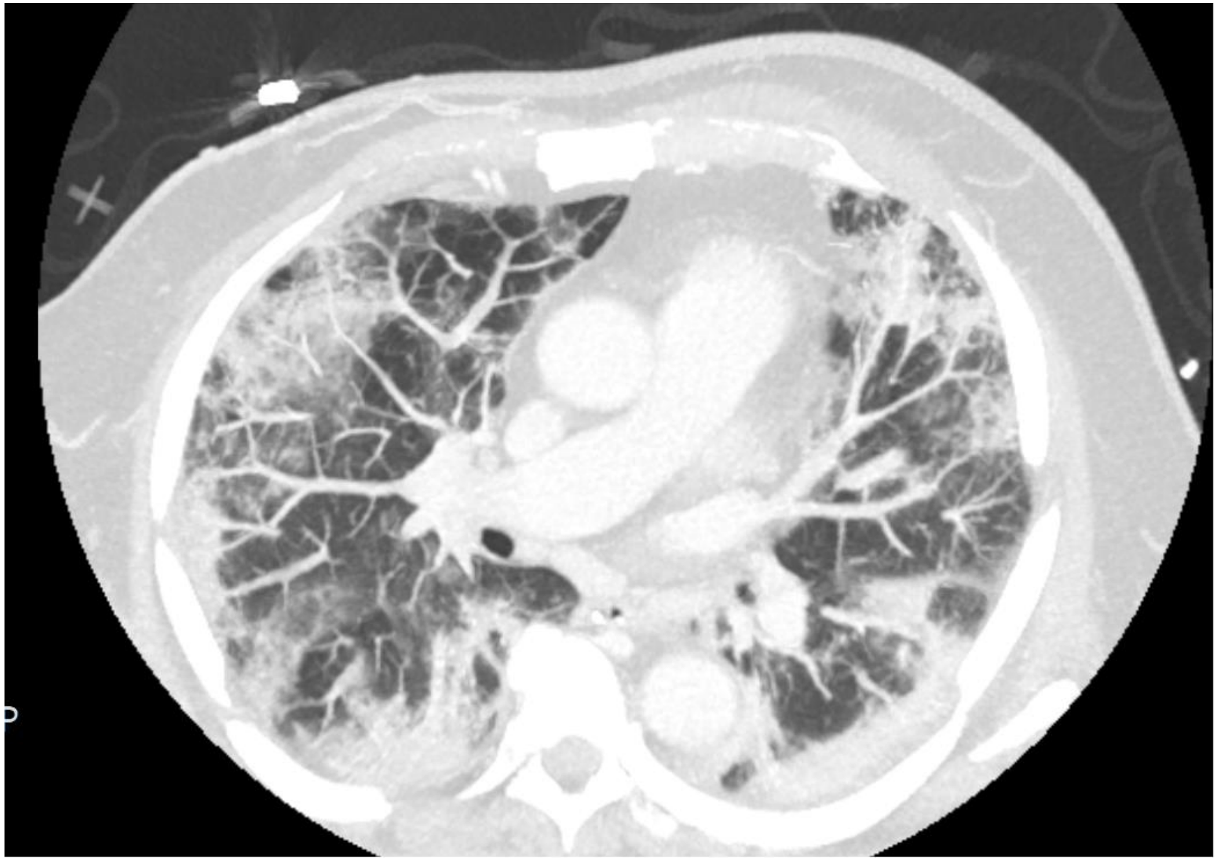

Supplement Figure 1: Patient N°4, CT Scan of the lung at LE, after first week of ICU stay due to COVID-19 associated ARDS (mechanically ventilated).
